# Oxidation of Organic Compounds Using Water as the Oxidant with H_2_ Liberation Catalyzed by Molecular Metal Complexes

**DOI:** 10.1021/acs.accounts.2c00328

**Published:** 2022-07-26

**Authors:** Sayan Kar, David Milstein

**Affiliations:** Department of Molecular Chemistry and Materials Science, The Weizmann Institute of Science, 234 Herzl Street, Rehovot 7610001, Israel

## Abstract

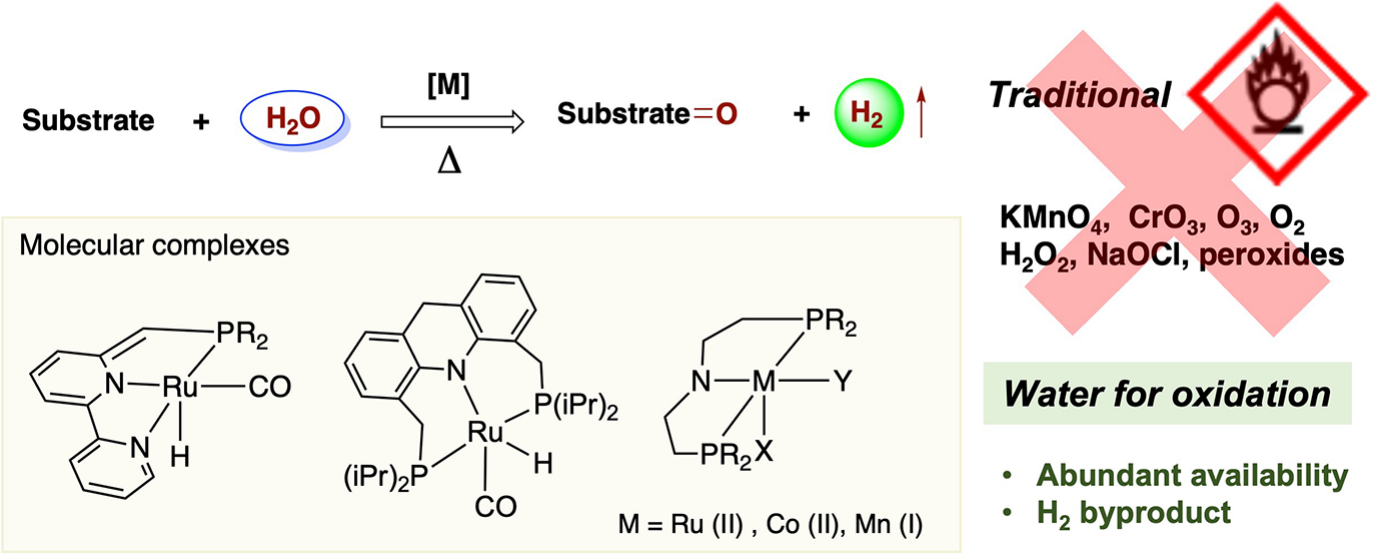

Oxidation reactions of organic compounds play a central role in both industrial chemical and material synthesis as well as in fine chemical and pharmaceutical synthesis. While traditional laboratory-scale oxidative syntheses have relied on the use of strong oxidizers, modern large-scale oxidation processes preferentially utilize air or pure O_2_ as an oxidant, with other oxidants such as hydrogen peroxide, nitric acid, and aqueous chlorine solution also being used in some processes. The use of molecular oxygen or air as an oxidant has been very attractive in recent decades because of the abundance of air and the lack of wasteful byproduct generation. Nevertheless, the use of high-pressure air or, in particular, pure oxygen can lead to serious safety concerns with improper handling and also necessitates the use of sophisticated high-pressure reactors for the processes.

Several research groups, including ours, have investigated in recent times the possibility of carrying out catalytic oxidation reactions using water as the formal oxidant, with no added conventional oxidants. Along with the abundant availability of water, these processes also generate dihydrogen gas as the reaction coproduct, which is a highly valuable fuel. Several well-defined molecular metal complexes have been reported in recent years to catalyze these unusual oxidative reactions with water. A ruthenium bipyridine-based PNN pincer complex was reported by us to catalyze the oxidation of primary alcohols to carboxylate salts with alkaline water along with H_2_ liberation, followed by reports by other groups using other complexes as catalysts. At the same time, ruthenium-, iridium-, and rhodium-based complexes have been reported to catalyze aldehyde oxidation to carboxylic acids using water. Our group has combined the catalytic aqueous alcohol and aldehyde oxidation activity of a ruthenium complex to achieve the oxidation of biomass-derived renewable aldehydes such as furfural and 5-hydroxymethylfurfural (HMF) to furoic acid and furandicarboxylic acid (FDCA), respectively, using alkaline water as the oxidant, liberating H_2_. Ruthenium complexes with an acridine-based PNP ligand have also been employed by our group for the catalytic oxidation of amines to the corresponding lactams, or to carboxylic acids via a deaminative route, using water. Similarly, we also reported molecular complexes for the catalytic Markovnikov oxidation of alkenes to ketones using water, similar to Wacker-type oxidation, which, however, does not require any terminal oxidant and produces H_2_ as the coproduct. At the same time, the oxidation of enol ethers to the corresponding esters with water has also been reported. This account will highlight these recent advances where water was used as an oxidant to carry out selective oxidation reactions of organic compounds, catalyzed by well-defined molecular complexes, with H_2_ liberation. The oxidation of alcohols, aldehydes, amines, alkenes, and enol ethers will be discussed to provide an outlook toward other functional groups’ oxidation. We hope that this will aid researchers in devising other oxidative dehydrogenative catalytic systems using water, complementing traditional oxidative processes involving strong oxidants and molecular oxygen.

## Key References

- BalaramanE.; KhaskinE.; LeitusG.; MilsteinD.Catalytic transformation of alcohols to carboxylic acid salts and H2 using water as the oxygen atom source. Nat. Chem.2013, 5, 122–12510.1038/nchem.153623344447.^1^*Water was used as an oxidant for the oxidation of primary alcohols to carboxylate salts while liberating H*_2_*gas, catalyzed by a ruthenium bipyridine-based pincer complex*.
- KhusnutdinovaJ. R.; Ben-DavidY.; MilsteinD.Oxidant-Free Conversion of Cyclic Amines to Lactams and H2 Using Water As the Oxygen Atom Source. J. Am. Chem. Soc.2014, 136, 2998–300110.1021/ja500026m24521458.^2^*The oxidation of cyclic amines to lactams was reported using water, catalyzed by a ruthenium acridine PNP-based pincer complex. H_2_ as the only byproduct of the reaction*.
- TangS.; Ben-DavidY.; MilsteinD.Oxidation of Alkenes by Water with H2 Liberation. J. Am. Chem. Soc.2020, 142, 5980–598410.1021/jacs.0c0159232182050PMC7118709.^3^*Markovnikov oxidation of alkenes to corresponding ketones was reported using water as an oxidant while liberating H_2_ gas, catalyzed by a ruthenium catalyst and a Lewis acid cocatalyst.*
- KarS.; ZhouQ.-Q.; Ben-DavidY.; MilsteinD.Catalytic Furfural/5-Hydroxymethyl Furfural Oxidation to Furoic Acid/Furan-2,5-dicarboxylic Acid with H2 Production Using Alkaline Water as the Formal Oxidant. J. Am. Chem. Soc.2022, 144, 1288–129510.1021/jacs.1c1090835007419PMC8796234.^4^*A homogeneous ruthenium complex was employed as a catalyst for the oxidation of biomass-derived furfural and HMF to valuable products, using alkaline water while generating H_2_*.

## 1 Introduction

**Figure 1:**
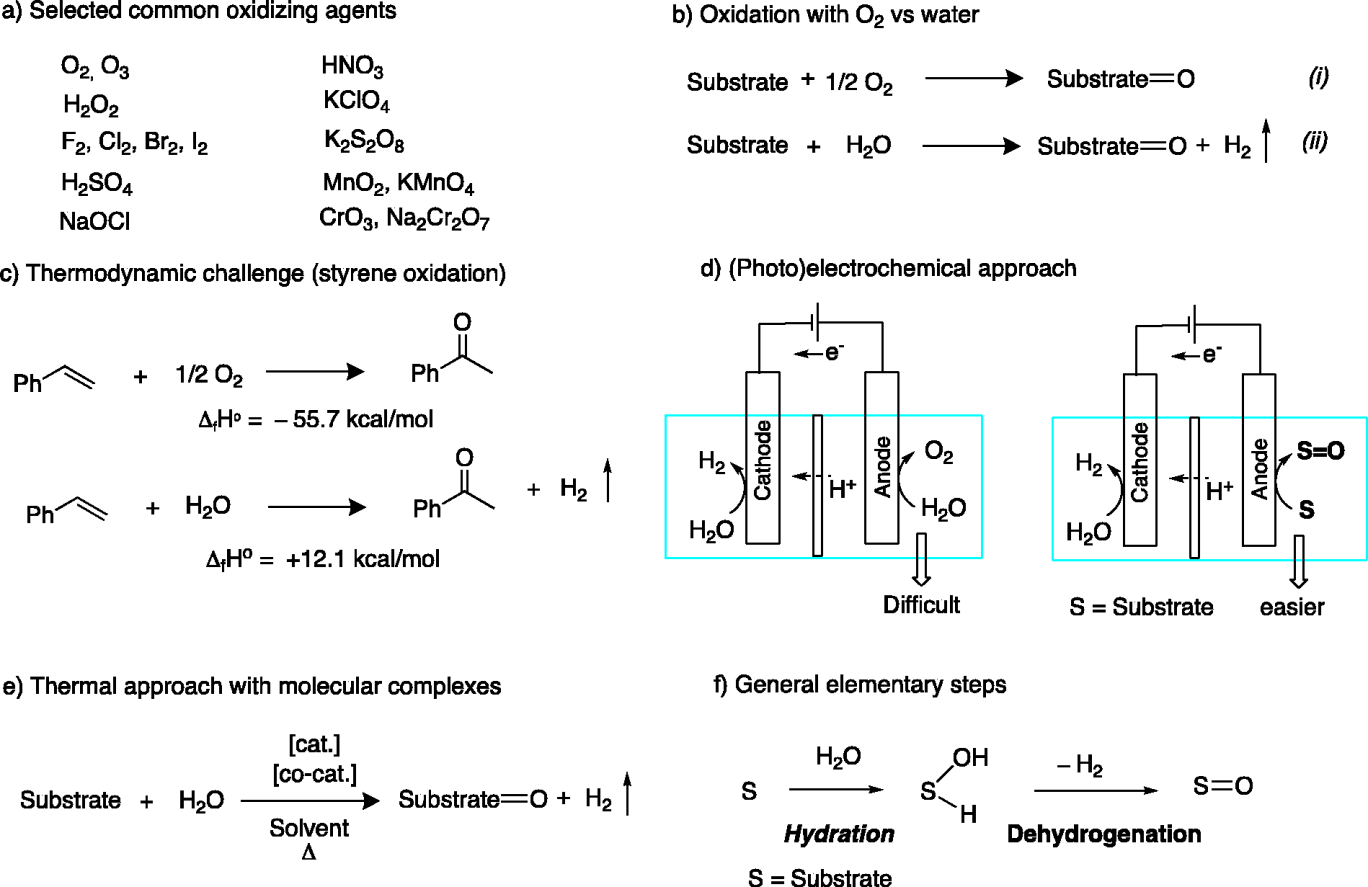
Oxidation of organic compounds using water as an oxidant vs other oxidants. (a) Selected common oxidizing agents, (b) oxidation with water as oxidant as compared to oxidation with molecular oxygen, (c) thermodynamic difficulty associated with achieving oxidation reactions using water, (d) the development of (photo)electrochemical systems for oxidation with water by replacing the kinetic bottleneck water oxidation with organics oxidations, (e) the thermal approach based on well-defined molecular catalysts, and (f) general elementary steps of hydration and dehydrogenation in oxidations with molecular complexes, in which the hydration step is often preceded by an additional dehydrogenation step which is not shown here.

The oxidation of organic compounds is central to the laboratory-scale synthesis of numerous fine chemicals and the industrial synthesis of many bulk chemicals.^5−7^ The traditional oxidation processes involve the use of high-energy strong oxidizers such as permanganates, dichromates, peroxides, halogens, metal salts, and others, which are energy-intensive to prepare, can be difficult to handle, and provide low atom economy while generating a large amount of reaction waste (Figure 1a). Modern oxidation methods have seen considerable progress over the last decades focusing on lessening the environmental impact by using greener reagents and solvents along with process optimization.^8^ The use of low-atom-economy strong oxidants is avoided, favoring the use of air as an oxidant whenever possible, followed by the use of O_2_.^9−14^ The aerobic oxidation is obviously favored by the abundance of air, which makes it economically advantageous, and it is generally safer than the use of O_2_ under pressure. Additionally, alternative oxidation protocols instead of traditional thermal processes, such as photo- and electrooxidation, are also being investigated.^15−17^

The intention of this Account is to highlight the use of water as an oxidant for the selective oxidation of organic compounds under mild thermal conditions. In contrast to the oxidations carried out using air or O_2_, which sometimes produce water as the reaction byproduct, oxidation with water produces H_2_ gas as the coproduct, which is a very valuable commodity and has been dubbed the fuel of the future (Figure 1b). Combining the cheap abundance of water with valuable H_2_ generation contributes to the economics of these transformations. Carrying out oxidations with water is challenging thermodynamically because of the stability of water and the high energy nature of H_2_ (Figure 1c). Nevertheless, molecular catalysts reported in recent years can carry out these oxidations under moderate temperatures (between 100 and 150 °C), driven entropically by the evolved H_2_, overcoming the challenge.

The use of water for oxidation is traditionally limited to the steam-reforming process of selected hydrocarbons that have been utilized industrially to produce H_2_ fuel along with CO or CO_2_.^18−20^ The steam reforming is typically limited to a handful of substrates, which are highly endothermic and require high temperatures, generally catalyzed by heterogeneous catalysts, and not useful for the preparation of oxidized organic products and so will not be discussed here. Over the past decade, two new approaches can be observed in the literature for processes that result in the oxidation of organic substrates using water under much milder conditions while simultaneously generating H_2_ gas. One of the approaches, based on (photo)electrochemical or photochemical reactions, is inspired by the water-splitting reaction, where proton reduction is coupled to organic oxidation to bypass the energy-intensive and often rate-limiting water oxidation step (Figure 1d). In recent years, several organic substrates have been oxidized using this approach, including alcohols, aldehydes, and waste materials such as cellulose and plastic.^21−25^ In a markedly different approach, various well-defined molecular metal complexes have also been reported recently that catalyze the oxidation of different substrates using water as the reactant while generating dihydrogen gas (Figure 1e). These complexes generally dissolve in the employed reaction medium to homogeneously carry out the oxidation. The reported oxidations of this kind can be mechanistically broken down, for the sake of simplicity, into the elemental steps of hydration and dehydrogenation (Figure 1f). In the majority of the reactions, the employed metal complexes catalyze the dehydrogenation of the substrates or intermediates,^26−28^ whereas the hydration step occurs either spontaneously or by an additional Lewis acid/base cocatalyst. Clearly, for such an approach to be effective, the working molecular catalysts should withstand the presence of water, which has greatly limited the number of molecular catalysts that can be used in such processes. Nevertheless, molecular complexes have been reported in recent times that not only are active in water but often show a rate enhancement in their catalytic dehydrogenation activities in the presence of water compared to when no water is present.

In this Account, we will discuss the organic (nonphotochemical or electrochemical) transformations that have been achieved using water as the oxidant with simultaneous H_2_ generation, catalyzed by molecular complexes. We will initially discuss the dehydrogenative oxidation of primary alcohols to carboxylic acids using water, first reported by our group and followed by other research groups. The reforming of small alcohols such as methanol, ethanol, and ethylene glycol catalyzed by various molecular complexes will also be described. Subsequently, we shall explore the aqueous aldehyde oxidation and how its combination with alcohol oxidation has allowed for the development of the oxidation of biomass-derived substrates furfural and 5-hydroxymethylfurfural (HMF). Subsequently, the oxidation of amines using water will be described, catalyzed by Ru acridine-based PNP pincer complexes, leading to the generation of lactones or carboxylic acids. At the end, we will focus on the oxidation of alkenes and enol ethers to the corresponding ketones and esters using water, without the requirement of a terminal oxidant and associated with H_2_ liberation, before concluding with a brief discussion of the possible future directions in this research area.

## 2 Oxidation of Primary Alcohols to Carboxylic Acids with Water

**Figure 2:**
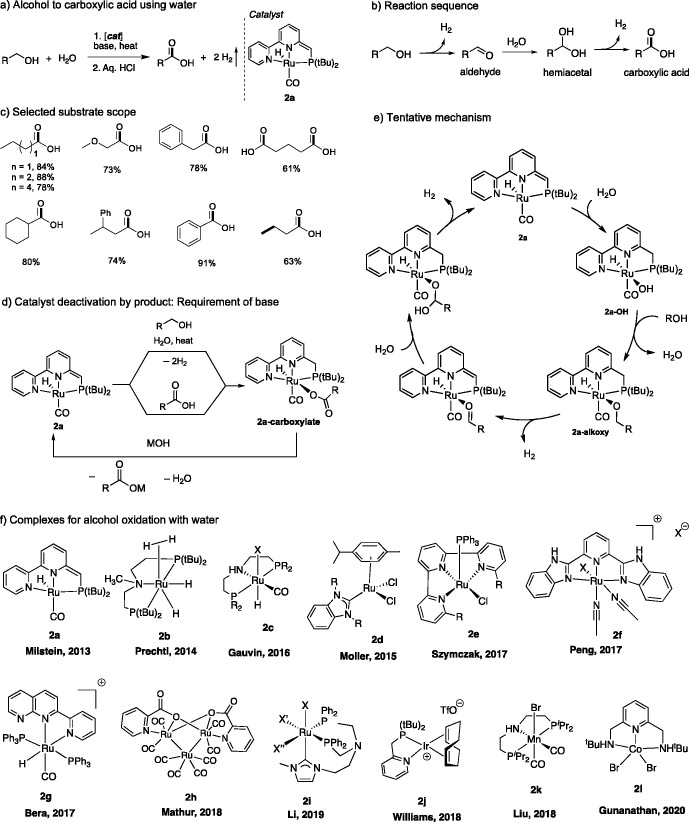
Oxidation of primary alcohols to carboxylic acids using water homogeneously catalyzed by metal complexes with H_2_ liberation. (a) Overall reaction with an initial report using a Ru-PNN complex, (b) reaction sequence from alcohol to acid, (c) selected substrate scope for oxidation, (d) requirement of a stoichiometric base to stop catalyst deactivation, (e) proposed reaction mechanism for the oxidation, and (f) a compilation of molecular catalysts reported for the transformation.

The oxidation of primary alcohols to the corresponding carboxylic acids is a fundamental reaction in organic chemistry, traditionally carried out by strong stoichiometric oxidizers such as KMnO_4_ and CrO_3_ in the laboratory. Current methods allow the oxidation to proceed using O_2_, although a majority of alcohol oxidations to acids are still carried out using toxic strong oxidants such as chromates and iodates with catalytic ruthenium oxide or in two steps via aldehyde intermediates. In 2013, we reported a direct alcohol oxidation procedure to carboxylate salts in alkaline water where water itself acts as the oxidant while producing H_2_ gas as a byproduct.^1^ The reaction is catalyzed by ruthenium PNN pincer complex **2a** (0.1 mol %) (Figure 2a) and proceeds without the requirement of any sacrificial hydrogen or oxygen acceptors, unlike previous reports.^29−31^ The elementary steps in operation during the reaction are as follows: The alcohol is initially dehydrogenated by the molecular complex, generating an aldehyde and one molecule of H_2_ (Figure 2b). Under these conditions, water’s attack of the aldehyde intermediate generates a hemiacetal intermediate, which is further dehydrogenated to produce the carboxylic acid and a second equivalent of H_2_ (Figure 2b). Under optimized conditions, several aliphatic and aromatic alcohols were converted to acids and H_2_ in moderate to high yields in the presence of catalytic **2a** (0.2 mol %) by refluxing the reaction mixture in an open system at 110 °C (bath temperature) under a flow of argon (Figure 2c). Importantly, only small amounts of acid were observed in the absence of base because of the binding of the acid product across the dearomatized Ru complex, leading to the generation of a deactivated carboxylate complex (Figure 2d). In the presence of base, the initially formed acid product is trapped, opening the metal center for catalytic turnover, while generating the carboxylate salt product. On the basis of experimental evidence and DFT calculations, we proposed a mechanistic cycle involving the formation of hydroxy (**2a**-OH), alkoxy (**2a**-alkoxy), and hemiacetoxy complexes as reaction intermediates in the catalytic cycle, where metal–ligand cooperation via dearomatization–aromatization of the central pyridine ring played a crucial role (Figure 2e).

Subsequently, other molecular complexes have also been employed for the aqueous oxidation of alcohols to the corresponding carboxylic acid salts (Figure 2f).^32,33^ For instance, the research groups of Prechtl^34^ and Gauvin^35^ have independently employed ruthenium PNP complexes for the oxidation of alcohols to carboxylic acid salts using water with good acid yields. Among the notable differences, the molecular complexes employed by Prechtl and co-workers (**2b**) featured a methyl moiety at the central N donor which barred metal–ligand cooperation in the system. The resulting complex still catalyzed the oxidation, although its catalytic activity was significantly lower compared to that of analogous complexes where MLC was present, as in the report of Gauvin and co-workers (**2c**). Similarly, Møller and co-workers have reported a tetrahedral Ru(II) complex with one NHC ligand (**2d**) to achieve the oxidation of alcohols to acids.^36,37^ In this case also, the lower catalytic activity necessitated a relatively higher catalyst loading (1 mol %) that was likely due to the absence of MLC in the system. The research groups of Szymczak,^37,38^ Peng,^38^ Bera,^39^ Mathur,^40^ and others have also reported different ruthenium-based complexes (**2e**–**2h**) for the selective oxidation of alcohols to acids using water, as shown in Figure 2f. Li and co-workers have reported an interesting ruthenium complex with an unusual facial coordination of a pincer ligand (**2i**), which showed high activity in the transformation.^41^ Other than ruthenium, Williams and co-workers have employed an Ir(I) complex (**2j**) for the oxidation, with a bidentate PN ligand.^42^ Among first-row transition-metal complexes, manganese-^43^ and cobalt^44^-based complexes (**2k** and **2l**) were successfully employed for alcohol oxidation, albeit with the expected catalytic activities being lower than those of their noble metal counterparts.

Notably, these transformations utilize a basic reaction medium, which is necessary in most cases because of the deactivating nature of the product carboxylic acid by coordination to the metal center. Nonetheless, the oxidation of benzylic alcohols to the corresponding carboxylic acids with water under base-free reaction conditions has also been demonstrated by Fujita, Yamaguchi, and co-workers using a dicationic iridium catalyst bearing a functional N-heterocyclic carbene ligand.^45^

### 2.1 Synthesis of Amino Acids

**Figure 3:**
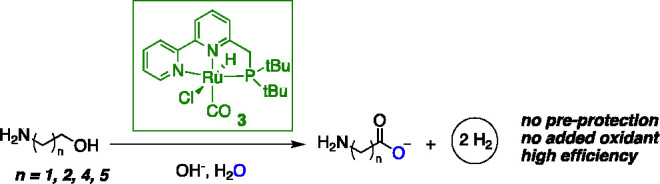
Synthesis of amino acids by the dehydrogenation of amino alcohols.

We have employed the alcohol oxidation protocol to develop a general method for the synthesis of amino acids by the oxidation of corresponding readily available amino alcohols by water (Figure 3),^46^ which previously often required the use of stoichiometric oxidants along with protection–deprotection steps. The major challenge in accessing the amino acids from amino alcohols was the possible self-coupling of amino alcohols, either intramolecular or intermolecular, generating lactams or amides as products. Under the optimized conditions, however, using ruthenium complex **3** as the catalyst (0.2 mol %) in a sufficiently basic solution, the formation of amino acid was favored, resulting in the synthesis of several naturally occurring amino acids including glycine, phenyl alanine, proline, and others in excellent yields with H_2_ production. The highly caustic reaction medium, however, results in the racemization of the α position even when an optically pure amino alcohol was used as the substrate.

### 2.2 Reforming of Small Alcohols for H_2_ Generation

**Figure 4:**
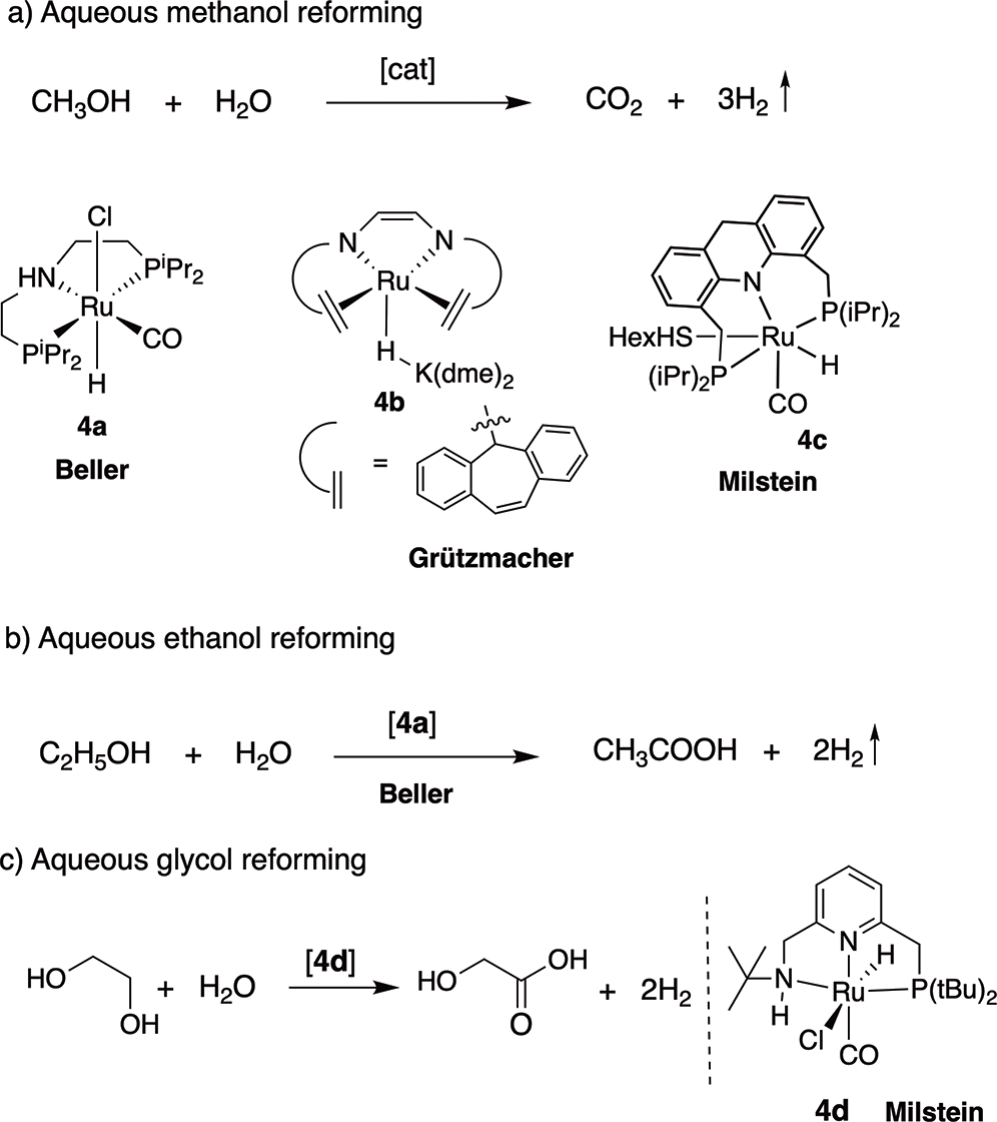
H_2_ generation from (a) methanol, (b) ethanol, and (c) an ethylene glycol–water mixture catalyzed by molecular complexes.

The catalytic activity of molecular complexes has also been utilized for the reforming of alcohols to generate H_2_ and an oxidation product from an alcohol/water mixture.^47−49^ The research groups of Beller^50,51^ and Grützmacher^51^ in 2013 independently reported ruthenium complexes for producing a CO_2_/H_2_ gas mixture via aqueous methanol reforming (Figure 4a). The reported molecular complexes were found to be active at much lower temperatures (90–100 °C) than for the heterogeneous catalysts that were used previously for the reforming (>200 °C). The high activity was attributed to metal–ligand cooperation, which was redox-innocent in case of the Beller system and noninnocent in the Grützmacher system. Subsequently, many complexes have been employed for aqueous methanol reforming, which have been summarized elsewhere in recent times.^49^ While most of the reported complexes require a strongly alkaline solution to function, base-free aqueous methanol reforming in a neat methanol/water mixture has also been demonstrated by us with high turnover numbers (>130 000) and frequencies (>600 h^–1^) using a ruthenium pincer complex (**4c**, Figure 4a).^52^ Similar to methanol, ethanol reforming catalyzed complex **4a** was also reported by Beller and co-workers^53^ and very recently by Zheng and co-workers using a ruthenium alkylidene complex (Figure 4b).^54^ Our group has also recently shown the reforming of ethylene glycol to glycolic acid with a ruthenium PNNH complex (**4d**) as a catalyst at mild temperatures (Figure 4c).^55^

## 3 Oxidation of Aldehydes to Carboxylic Acids Using Water

**Figure 5:**
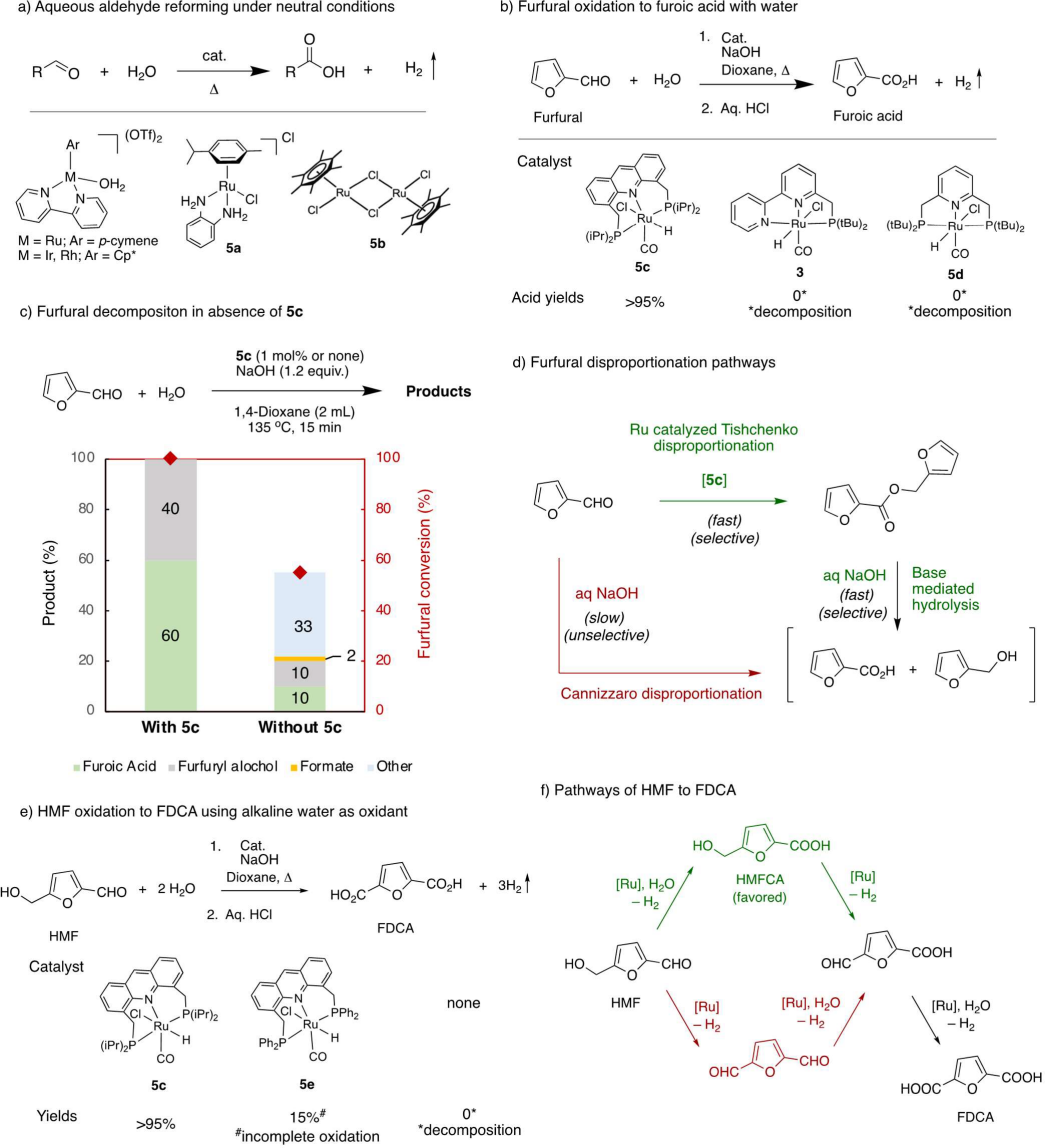
Oxidation of aldehydes using water. (a) Aqueous aldehyde reforming to the corresponding acids using molecular complexes. (b) Oxidation of biomass-derived furfural to furoic acid with water catalyzed by Ru-PNP complex **5c**. (c, d) Active involvement of [Ru] in suppressing furfural decomposition via an induced disproportionation pathway. (e) HMF oxidation to FDCA using water with H_2_ liberation. (f) Pathways for HMF oxidation to FDCA.

The oxidation of aldehydes to carboxylic acids in water liberating H_2_, also known as the aldehyde–water shift reaction, is traditionally carried out using heterogeneous catalysts at very high temperatures (>200 °C). Whereas the previously discussed alcohol oxidation reaction using water catalyzed by molecular complexes is surmised to proceed via an aldehyde intermediate, carrying out the oxidation using an aldehyde as the substrate is challenging because aldehydes are prone to degradation under basic conditions. At the same time, a high concentration of the aldehyde can deactivate the complexes via the formation of off-cycle intermediate complexes.^56,57^ Aldehyde oxidation to carboxylic acid by water catalyzed by RuH_2_(PPh_3_)_4_ was reported by Murahashi and co-workers in 1987 in the presence of phenylacetone as an H_2_ acceptor, with ester formation being favored in the absence of the H_2_ acceptor.^58^ Stanley and co-workers in 2004 reported a homogeneous dirhodium tetraphosphine complex that can oxidize acetaldehyde and heptanal to their corresponding acids using water under acceptorless conditions.^59^ In 2014, Cundari, Heinekey, and co-workers reported iridium, rhodium, and ruthenium-based half-sandwich complexes for aldehyde oxidation to carboxylic acids under neutral conditions at 0.4–0.8 mol % catalyst loading and a 105 °C reaction temperature (Figure 5a), simultaneously releasing H_2_ gas.^60^ While the Ir and Rh complexes showed high catalytic activity, the Ru complexes were significantly less active. However, the substrate scope was found limited, with only acetaldehyde, propionaldehyde, *tert*-butaldehyde, and benzaldehyde being amenable to the oxidation to a significant extent. In 2016, Goldberg and co-workers reported improved ruthenium complexes (**5a**) for the aldehyde–water shift reaction, which displayed a broader substrate scope, nevertheless being limited to aliphatic aldehydes (Figure 5a).^61^ The same group in 2021 reported hexamethylbenzene-based ruthenium complexes (**5b**) which were more active in the reaction.^62^ Still, relatively low catalytic activities and a narrow substrate scope were observed, which in part can be attributed to the deactivating nature of the product carboxylic acid.

### 3.1 Oxidation of Biomass-Derived Aldehydes with Water

Biomass-derived renewable aldehydes, specifically, furfural and 5-hydroxymethyl furfural (HMF), represent important chemical feedstocks that are expected to be crucial in the coming years for the sustainable production of commodity chemicals.^63,64^ For example, 2,5-furandicarboxylic acid (FDCA), which can be synthesized via the complete oxidation of HMF or from furfural via oxidation and carboxylation, is a chemical that can potentially replace terephthalic acid in polymer synthesis. Our group has recently reported molecular complexes that can catalyze the oxidation of furfural to furoic acid using alkaline water as the oxidant while liberating H_2_ gas (Figure 5b).^4^ The reaction proceeds at 135 °C with 1 mol % catalyst (**5c**) loading and produces furoic acid and H_2_ in high yields (>95%). The acridine-based Ru-PNP pincer complex **5c** is uniquely active for this oxidation process because when other pincer complexes were employed, unselective decomposition of the substrate was observed. Our mechanistic investigation revealed that the employed complex **5c** facilitates the disproportionation of the furfural substrate prior to the onset of decomposition, which is critical for obtaining the oxidation product in high yields (Figure 5c,d). The practicality of the system was demonstrated by developing a catalyst recycling scheme as well as by conducting a gram-scale reaction.

We further combined the alcohol oxidation and aldehyde oxidation with water to directly obtain useful chemical FDCA and H_2_ from an HMF/alkaline water mixture (Figure 5e). When precatalyst **5c** (2 mol %) and HMF were held in an alkaline water–dioxane solvent mixture at 160 °C for 68 h, FDCA was obtained in >95% yield along with producing a nearly quantitative amount of H_2_. Significantly, upon changing the phosphine substituent of the precatalyst from isopropyl to phenyl, the FDCA yield decreased drastically while forming the partially oxidized product 5-hydroxymethylfuroic acid (HMFCA) in 70% yield. This also suggests that under the reaction conditions, the aldehyde group is oxidized prior to the alcohol groups and oxidation to FDCA proceeds via the HMFCA intermediate (Figure 5f). The high activity and selectivity of the catalyst along with the high working substrate concentration seem promising for the scale-up of the process.

## 4 Oxidation of Amines by Water

Compared to the oxidation of alcohols, the oxidation of amines using water as an oxidant has received far less attention. This is because while many catalysts are active in catalyzing alcohol dehydrogenation to aldehydes, few complexes can catalyze the amine dehydrogenation. Moreover, the requirement of a water-resistant nature, which is necessary for the utilization of water as an oxidant, makes aqueous amine oxidation further challenging.

### 4.1 Cyclic Amine Oxidation to Lactams

**Figure 6:**
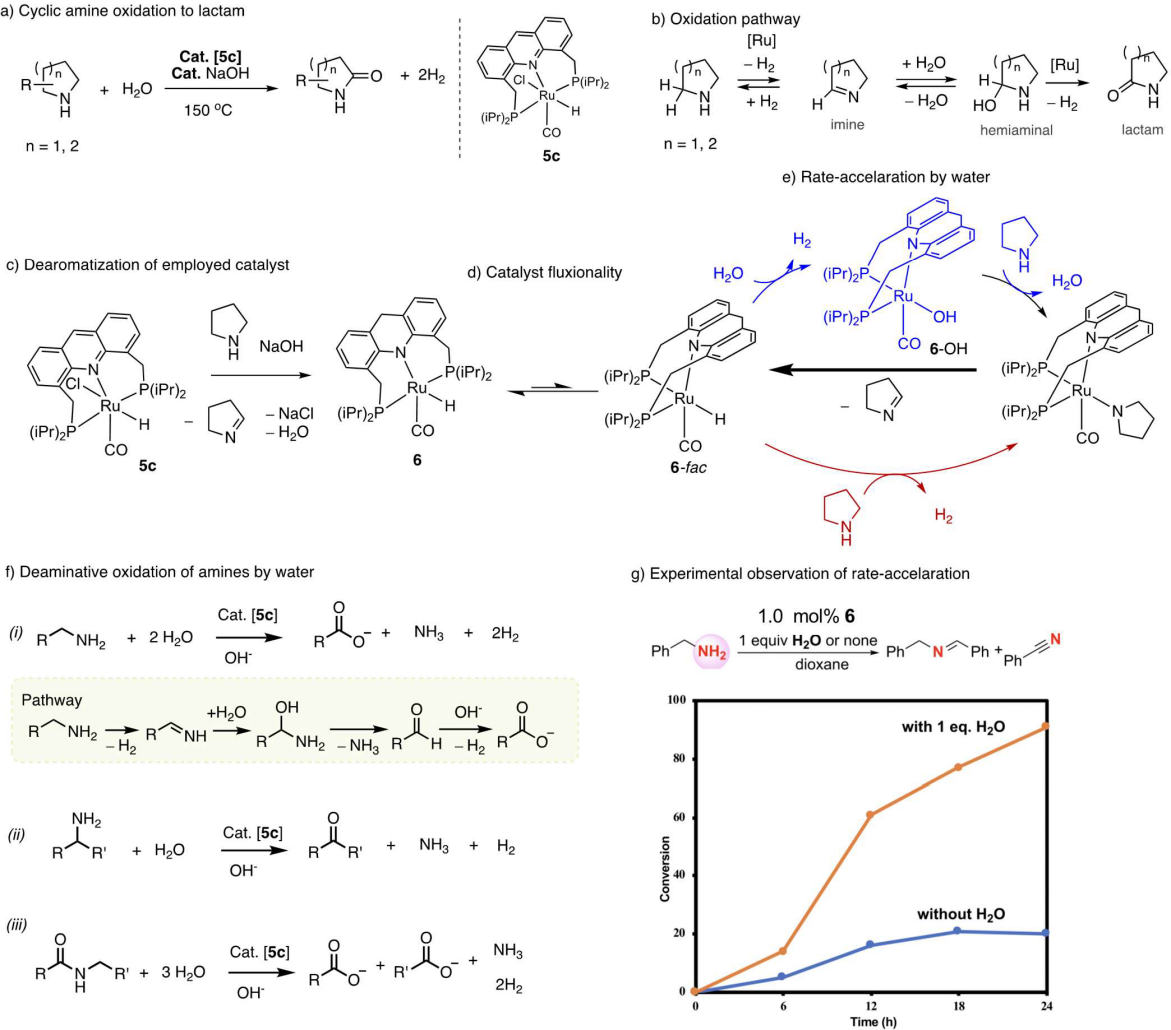
Oxidation of amines using water with H_2_ liberation. (a) Oxidation of secondary cyclic amines to lactams with water, (b) pathway to lactam formation, (c) initial dearomatization of the employed molecular complex, (d) fluxionality between the facial and meridional coordination mode of the ligand in an *in situ* generated catalyst, with the catalysis occurring via *fac* coordination, (e, g) rate enhancement in amine dehydrogenation in the presence of water, and (f) the deaminative oxidation of amines and amides to carboxylates or ketone under basic conditions catalyzed by ruthenium acridine-based complexes.

We have recently reported that the acridine complex **5c** possesses the two desirable properties of being active in amine dehydrogenation and also being catalytically active in water.^65^ Consequently, we discovered that complex **5c** can catalyze the direct oxidation of cyclic amines to lactams using water while liberating 2 equiv of H_2_ gas (Figure 6a).^2^ The α-methylene unit is converted to a C=O unit during the transformation, which has previously required the use of strong oxidizing reagents. The pathway of amine to lactam formation can be simplified in three steps involving initial amine dehydrogenation to imine, followed by water addition to the C=N bond to generate a hemiaminal intermediate with the final step being hemiaminal dehydrogenation to lactam (Figure 6b). The reaction requires a catalytic amount of base to convert the employed precatalyst **5c** to the actual catalyst. Mechanistic investigations revealed that in the presence of base and cyclic amine, the aromatic complex **5c** is first converted to a dearomatized complex **6**, which then catalyzes the reaction (Figure 6c).^66,67^ Consequently, the dearomatized complex **6** catalyzes the reaction even under neutral conditions in the absence of base. Important insights regarding the catalytic cycle were obtained by DFT studies. It was found that while the most thermodynamically stable form of complex **6** features the ligand bound in a meridional fashion, the complex is nonetheless fluxional, with the *fac* isomer facilitating its catalytic activity (Figure 6d). The *fac* isomer was calculated to be 9.1 kcal/mol higher in energy than the *mer* isomer.^67^ Interestingly, the DFT studies indicate that two distinct mechanisms are possible for the initial amine dehydrogenation. The first is N–H activation and H_2_ liberation, followed by β-hydride elimination, which can proceed without water (Figure 6e). However, in the presence of water, a water-assisted route opens up involving the generation of a *fac* hydroxy intermediate (**6**-OH), which in turn assists the amine dehydrogenation (Figure 6e). DFT calculations suggest that the water-assisted route is more facile, which was later also verified experimentally in a separate study (as explained later). The hydration of the generated imine was calculated to be spontaneous, with a ruthenium-assisted pathway significantly higher in energy. The final step of the reaction, which is the hemiaminal dehydrogenation, was found to be facile under the reaction conditions, involving the *fac* isomer. A variety of cyclic amines, involving pyrrolidines, piperidine, and morpholine, can be oxidized using this procedure to their corresponding lactones. For linear diamines, only minor amounts of amide were observed as a result of its hydrolysis to the aminoalcohol intermediate, followed by its cyclization to form a cyclic amine.^65^

### 4.2 Amine Oxidation to Carboxylates/Ketones with Water

We have recently demonstrated that by using the molecular complex **5c** as a catalyst, linear primary amines can be oxidized to their corresponding carboxylate salts under alkaline conditions (Figure 6f-i).^68^ The reaction is associated with the liberation of 2 equiv of H_2_ and 1 equiv of ammonia and proceeds in the presence of 1 mol % **5c** and a stoichiometric amount of NaOH base at 150 °C (48 h). Carboxylates in high yields were observed under these reaction conditions for a variety of linear primary amines, including both aliphatic and aromatic amines. The pathway to obtaining a carboxylic acid from the amine consists of the initial dehydrogenation of the amine to imine by [Ru], followed by deaminative hydration of the imine to generate aldehyde via a hemiaminal intermediate, with the final step being the aldehyde oxidation by water while liberating H_2_ (Figure 6f). Similar to the previous study, the actual catalyst is dearomatized complex **6** which is generated *in situ*, and the dehydrogenation reactions proceed via the involvement of the *fac* isomer. The first amine dehydrogenation to imine step is surmised to proceed in a more facile manner when water is present, via the formation of a ruthenium hydroxy intermediate. This was also experimentally verified, which showed a 4-fold increase in the acceptorless benzylamine dehydrogenation rate in the presence of 1 equiv of water (Figure 6g). Similar to linear terminal amines, α-branched primary amines can also be oxidized by water using **5c** as a catalyst, generating the corresponding ketones as products along with the formation of 1 equiv each of hydrogen and ammonia (Figure 6f-ii) Other than amines, when amides were used as substrates under the reaction conditions, the formation of 2 equiv of carboxylic acids was observed: one via the hydrolysis of the amide and the second via the subsequent oxidation of the released amine during hydrolysis (Figure 6f-iii).

## 5 Oxidation of Alkenes by Water

### 5.1 Oxidation of Alkenes to Ketones

**Figure 7:**
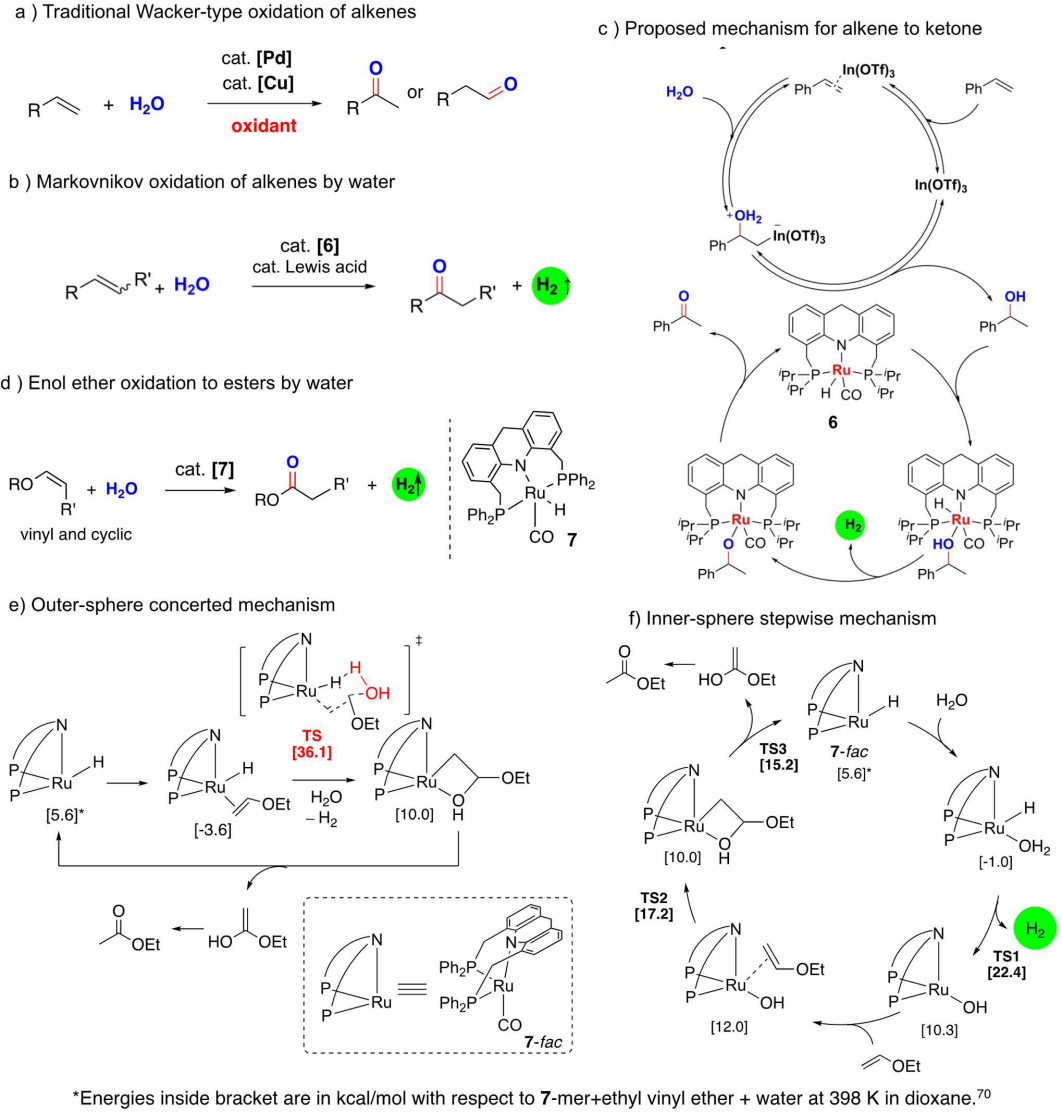
Oxidation of alkenes with water catalyzed by ruthenium complexes. (a) Traditional Wacker-type oxidation of alkenes with terminal oxidants, (b) ruthenium-catalyzed oxidation of alkenes with water while liberating H_2_ in the presence of a Lewis acid cocatalyst, (c) mechanism of alkene oxidation via tandem hydration–dehydrogenation, (d) additive-free oxidation of vinyl and cyclic enol ethers to esters using water, (e) concerted outer-sphere mechanism, which is less favorable, and (f) the more favorable inner-sphere stepwise mechanism involving the initial generation of a ruthenium hydroxy complex.

The oxidation of alkenes to the corresponding ketones and aldehydes is an important process, carried out industrially on a large scale. One of the most frequently employed processes for alkene oxidation is the Wacker process, which uses palladium complexes as catalysts and O_2_ as the terminal oxidant (with a copper salt as a mediator) (Figure 7a).^69^ The whole process uses an alkene and oxygen from the air as the reactants while producing the aldehyde/ketone and water as the products. We have recently reported that instead of air, water can be used as an oxidant for the oxidation of alkenes to ketones while liberating H_2_ gas (Figure 7b).^3^ The reaction is catalyzed by ruthenium acridine-based pincer catalyst **6** in conjunction with In(OTf)_3_ as a cocatalyst. In(OTf)_3_ catalyzes the initial hydration of the alkene to a secondary alcohol which subsequently undergoes Ru-catalyzed dehydrogenation to generate the desired ketone product (Figure 7c). Interestingly, although the initial hydration of styrene substrates to *sec*-alcohols is reversible, with the opposite reaction being favored, coupling this step with tandem dehydrogenation drives the reaction forward. Furthermore, the presence of both [Ru] and In(OTf)_3_ also suppresses the homocoupling of styrene, which is otherwise known to proceed in the presence of Lewis acids. When **6** (1.5 mol %) is used as the catalyst and In(OTf)_3_ is used as the cocatalyst (2–40 mol %), several styrene derivatives can be converted to the corresponding ketones in high yields. Although the monosubstituted terminal styrenes afforded good yields, disubstituted styrene with an internal alkene bond showed minimal reactivity likely because of the difficulty with hydration. Other than styrenes, strained double bonds such as in norbornene and cyclohexene can also be converted to the corresponding ketones. Interestingly, the ketone was observed to be the major oxidative product with no aldehyde formation, suggesting that under the conditions no anti-Markovnikov hydration takes place.

### 5.2 Oxidation of Enol Ethers (Vinyl and Cyclic) to Esters

As discussed earlier, the oxidation of alkenes using water requires a Lewis acid cocatalyst in addition to the ruthenium catalyst to carry out both the hydration and dehydrogenation steps. We have recently found that for specific alkenes which are electron-rich and easier to hydrate, such as enol ethers, the ruthenium complex itself can catalyze both steps leading to oxidation without any cocatalyst. Thus, when vinyl ethers are held at 125 °C in the presence of complex **6** (1.5 mol %) and water (0.25 mL) in dioxane, acetate esters are produced in moderate yields along with pure H_2_ gas (Figure 7d).^70^ The main side product of the reaction was an alkyl ethyl ether via hydrogenation of the substrate under the reaction conditions. Interestingly, the selectivity improved greatly when an analogue of the acridine complex with PPh_2_ ligands (complex **7**), bearing lower electron density at the metal center, was used, leading to >80% ester yields and <5% ether yields. Similar to vinyl ethers, cyclic enol ethers such as dihydrofuran and dihydropyran can also be oxidized to the corresponding lactones at 150 °C. However, noncyclic internal enol ethers were not amenable to oxidation because of their increased steric bulk around the double bond which inhibits their coordination to the metal center. Density functional theory studies yielded important insights regarding the reaction mechanism, suggesting an inner-sphere coupled dehydrogenation–hydration via the formation of a hydroxy intermediate complex rather than a concerted outer-sphere hydration–dehydrogenation mechanism (Figure 7e,f) or a tandem hydration–dehydrogenation pathway. The inner-sphere pathway was calculated to have an activation barrier of ∼26 kcal/mol compared to the outer sphere pathway (∼39 kcal/mol). It is to be noted that because the employed ruthenium catalyst is only weakly Lewis acidic, it requires an electron-rich substrate for effective oxidation with water, in the absence of any cocatalyst. Our group is currently looking for ways to increase the Lewis acidity at the metal center, without impeding its dehydrogenation reactivity, in order to increase the substrate scope toward unactivated alkenes.

## 6 Summary and Outlook

The oxidation of organic substrates using water as an oxidant while liberating H_2_ gas is a promising direction that can simultaneously address the sustainable generation of both our fuels and commodity chemicals if applied on a large scale. Although this new approach is thermodynamically more challenging than using strong oxidants, recent reports show that several molecular complexes display excellent catalytic activities to facilitate such transformations at moderate temperatures (100–150 °C). The oxidation of alcohols to carboxylic acids by water has been achieved under alkaline conditions with several molecular complexes as shown. At the same time, aldehyde oxidation to carboxylic acids, which is challenging because of complex deactivation pathways in the presence of aldehydes, has also been recorded. Our group has combined the alcohol and aldehyde oxidation with water to generate renewable polymer precursor FDCA along with pure hydrogen from a biomass-derived HMF and water mixture. Besides alcohols and aldehydes, catalytic amine oxidation with water has also been achieved, albeit with only a few molecular complexes, as a result of the limited number of complexes active in amine dehydrogenation. Using an acridine PNP-based ruthenium complex, our group has shown the dehydrogenative oxidation of cyclic amines to lactams using just water. At the same time, we have also shown that alkaline conditions can facilitate a deaminative oxidation pathway for amines to generate the corresponding carboxylate salts or ketones from an amine/water mixture. Among other substrates, molecular catalysts, in the presence of the In(OTf)_3_ cocatalyst, also facilitate the Markovnikov oxidation of alkenes to ketones using water but no other added oxidant while generating dihydrogen. This contrasts with the currently employed Wacker-type oxidations that require O_2_ as a terminal oxidant and produces water as a byproduct. The oxidation of enol ethers to esters using water has also been reported, using ruthenium complexes without any cocatalysts. Future efforts in this direction could be oriented to the oxidation of other functional groups with water using metal complexes. Coupled with the vast literature of dehydrogenation reactions, the water-based oxidation reactions can open the way for new green oxidative industrial processes.
